# Supplementary material for: Isolation, Identification, and Pathogenicity of a Goose Astrovirus Genotype 1 Strain in Goslings in China
Source: Viruses. 2024 Mar 30;16(4):541. doi: 10.3390/v16040541 (PMC11055043; doi:10.3390/v16040541)
Supplement: Supplementary file 1 [file viruses-16-00541-s001.zip › viruses-2810715-supplementary.pdf]

Table S1. Details of all isolated GAstV-1 strains

Virus strains	GenBank accession numbers	Length	Date	Location	Tissue
**FLX**	NC_034567	7299	2014	Hunan	liver
**AHDY**	MH410610	7288	2017	Anhui	liver、spleen、kidney
**TZ03**	MW353015	7262	2019	Jiangsu	Liver、Heart、kidney
**SCCD**	MW340534	7255	2019	Sichuan	liver
**ZJC14**	OK571391	7297	2020	Zhejiang	kidney
**JXYC**	OL762472	7310	2021	Jiangxi	embryo
**JXGZ**	OL762471	7310	2021	Jiangxi	embryo
**JSXZ**	OR827024	7299	2022	Jiangsu	kidney

Table S2 Primers used in this study for detection of the viruses

Primer name	Sequence (5′→3′)	gene	Product size (bp)
**TMUV F**	GCCACGGAATTAGCGGTTGT	E gene of duck Tembusu virus	401bp
**TMUV R**	TAATCCTCCATCTCAGCGGTGTAG		
**GHPV F**	GAGGTTGTTGGAGTGACCACAATG	VP1 gene of goose hemorrhagic polyomavirus	144bp
**GHPV R**	ACAACCCTGCAATTCCAAGGGTTC		
**GPVF**	AGACTTATCAACAACCATCAT(C) T	VP1 gene of goose parvovirus	779bp
**GPVR**	TCACTTATTCCTGCTGTAG		
**GRVF**	TGAGACGCCTGACTACGATT	S1 gene of goose reovirus	380bp
**GRVR**	ATGCTTGGAGTGAGACGACT		

**AIV**	GCCATCCTAGCAACGACTGT	NP gene of AIV	560bp
**AIV**	CCTGAAGTGCCACAAAATACAA		
**GAstV-1-F**	TGGTGCGAAAGGAGG	ORF1a gene of goose astrovirus genotype 1	564bp
**GAstV-1-R**	GGTTGACATAGCATAGCG		
**GAstV-2-F**	ATTCTTGGCTCGGTTGTC	ORF2 gene of goose astrovirus genotype 2	489bp
**GAstV-2-R**	CCTGTGTTGCTCCTTCTC		
